# Honoraria

**Published:** 1861-04

**Authors:** 


### Art. XI.—HONORARIA.

There is a highly pleasing idealism in the conception of an honorarium which should at all times be carefully cherished. On the other hand, so great an amplitude of realism enters into our notions of one that the least excursive mind rests gratefully upon the subject. In fact, abstractedly considered, an honorarium offers matter for much and profound reflection; while of the concrete question—alas! "'tis the general humour of the world; commodity steers our affections throughout."

If the subject be regarded from a philosophical aspect, we learn, as well from history as from observation, that the mind has not failed to deal with it as with every other question into which an ideal and a real element justly enters. As the thoughts have oscillated towards one or the other phase of the question, this has been invested now with a preponderance of idealism, now of realism; while scepticism and mysticism have not failed still further to discompose the vexed reason with their perturbing influence.

The differential character of an honorarium consists in its ideal element. Upon the right conception of this fact hinge the whole of the vexed questions connected with honoraria. We *pay* our servants or our tradespeople; we give an *honorarium* to our advocate or physician. In trade we render an accurately calculated return in money or substance for value received; in a profession the benefit rendered cannot be submitted to the ordinary rules of valuation, and a gift is of necessity substituted for a payment properly so called. In merchandise the *quid pro quo* is the same for the pauper as the millionaire; in professional service it varies partly according to the wealth of the receiver, partly according to the reputation of the giver, and for a like benefit the honorarium may be simply a grateful "thank you" (as is often, very often, the case), or a hundred or more guineas (as is very rarely the case).

The mental expenditure of a physician in any given case, is the essential thing of value which he yields to his patient. But in what manner could we arrive at a just estimate of the worth of this, so that the individual benefited by it could return due and fitting compensation for that benefit? The shrewdest business talent recoils perforce from the solution of this question, and our estimate of the equivalent to be rendered for professional aid must of necessity be based on very different and altogether secondary, but far from unimportant grounds. Thus, the gravity of the case attended, the time devoted to the care of the patient, and the reputation of the physician—all these things must enter into our consideration in endeavouring to calculate the importance of the service rendered. Hence it is that strictly speaking, the physician can never be paid an equivalent for the aid he renders; and in recognising and acting upon this truth, and renouncing voluntarily the right to demand legally any remuneration, does *honour* to himself; and the patient, acting upon the same truth, and presenting such a gift as his means afford, also does *honour* to himself; and such a gift is most properly termed an *honorarium.*

Now, sundry physicians entertain so lofty an idea of their science, that they will neither ask for a fee, however justly it may be due, nor specify the amount of one if asked so to do. Their souls revolt from the barest notion of anything that may have the semblance of trading out their talents in the ordinary forms of barter. "The physician who exclaims 'Give! Give!' when a sufferer cries 'Oh! Oh!' is worthy only to be a hangman or headsman." So said Theodore Zuinger, of Basil, and it is asserted that he would not even take a fee from the rich, except when forced upon him. "Madame," quoth a living and highly talented practitioner to a lady when, after a long and harassing attendance upon her daughter, she had asked him to state what she was indebted to him,—"Madame, I cannot make a charge." "Sir," she rejoined, "I am not so wealthy that I can give you a fee proportionate to my own estimate of the service you have rendered to my daughter, I must, therefore, at least beg of you to give me some idea of the amount of remuneration I ought to tender to you." "Madame," he said, "I should regret to offend you, but I cannot degrade my profession to the level of a trade. Whatever fee you think fit to give me I shall receive with satisfaction." "Sir,"

was the reply, "I cannot enter into any discussion with you upon questions of professional etiquette or honour. I am loth to wound your feelings, but it is impossible for me to look upon this matter otherwise than as one of simple business, in which I am prepared to pay what you conceive will be a just remuneration for such trouble as you have been at on my account, so far as that trouble admits of being remunerated. For the rest I can only offer you my gratitude." The practitioner smiled and bowed. It was evident that such a commonplace view of the matter would neither ruffle his feelings nor cause his principles to deviate in the slightest degree from their beaten track. Many months, indeed, passed before he was induced to give the lady any help in solving the difficulty, when a satisfactory honorarium was given to him, and —the medical charge of the lady's family given to another, who looked upon honoraria from a less ideal point of view. "I could not retain longer the services of the former gentleman with due respect to myself," said the lady, "or with the prospect of much longer maintaining any respect for him."

There are other physicians who look upon their profession simply as a means of earning substance, and who, in a thoroughly mercantile spirit, seek by the practice of it to obtain as large a pecuniary profit out of their patients as practicable. With these men the honorarium is the end and object of their labour. They chiefly admire the precepts of John of Gaddesden, who was accustomed to urge the fitness of physicians making sure of their emolument before taking a case in hand; they mostly follow the practice of M. Dumoulin, who would have his fee whenever and however often he saw a case. If a patient asked, "Doctor, shall I see you again?"—"Yes," he would answer, "if you pay me." "Is it necessary that I should pay you immediately?" "Certainly, if you wish to see me again." These are the physicians who, as Mosca phrases it in *Volpone*, "flay a man, before they kill him." Dr. Johnson states of his friend Levett, that "he would swallow what he did not like, nay, what he knew would injure him, rather than go home with an idea that his skill had been exerted without recompense; and had his patients maliciously combined to reward him with meat and strong liquors, instead of money, he would either have burst, like the dragon in the Apocrypha, through repletion, or been scorched up, like Portia, by swallowing fire." Very recently the public has been edified and the profession somewhat astonished by a fact which transpired in the progress of a law-suit, to the effect that a medical practitioner had claimed fees amounting to upwards of nine hundred pounds for a three-months' attendance upon a case which was not, so far as could be learned from the evidence, of the most exacting character.

These illustrations will show the typical modes in which the idealism and realism of honoraria are manifested. Fortunately, however, for the welfare of medical men and the public, a well-founded scepticism steps in, and, for the most part, restrains the ideal element of the honorarium within bounds compatible with the rough requirements of every-day life; while a certain mysticism, represented by a happy and enduring faith that medical science has a somewhat loftier aim than one of mere personal aggrandizement, holds in check alike both the scepticism and the realism, preventing the former becoming the mere tool of the latter. The majority of medical men, indeed, temper their ideal conceptions of the nobility of their profession with a proper regard to the requirements of commonplace existence. Thus, while, on the one hand, they yield without stint their aid to the poor, neither seeking for or requiring any honorarium from them, other than the thanks of a grateful heart; on the other hand, they look for such guerdon from the wealthier classes as might be anticipated from a generous appreciation of the important services rendered during sickness.

We frankly confess, even at the risk of being charged with an extravagancy of idealism, that the pleasantest reminiscences of our own professional career (one, be it said in all humility, by no means lacking experience) are those connected with the simple but warm gratitude of the poorer and poorest classes,—those who had nothing to offer but thanks, and those who, having but little, strove to force that little upon you, and make it much with the warm out-pourings of a thankful heart.

Certainly, the most enviable fee that ever we received was a *smile* from one who, with barely a rag to cover her, was laid upon a heap of filthy straw, in a cellar. We were passing along a bye-street in one of our great manufacturing towns, at a time when fear of "*the* Fever" had gone abroad. Several women were standing upon the causeway, talking earnestly. As we came up to the group, one of the women stepped up to us, and, curtseying, said, "I ask your pardon, sir, but please are you a doctor?" "Yes, my good woman," we replied; "can we help you anyway?" "Well, sir," she answered, "there was a woman and three children ill of fever down in that cellar (and she pointed to a narrow flight of steps littered with filth and offal), and we have seen nothing of them for three days. One of the children, a little girl, was the last person we saw up the steps, and she had been for some physic to the workhouse. The parish doctor saw her mother and the other children three or four days ago, but he has not been since. Me and my neighbours don't know what to make of the matter, and we were talking of it when you came up." "Then have none of you," we said, "been down into the cellar to see what was going on?" "O yes, sir," answered a woman, "I looked in a few moments ago, and saw that the mother and children were all very ill with the fever. But I dare not stay, for I have a young family, and so has each of my neighbours." And then came a chorus of requests that we should enter the cellar and help the poor woman and her children. "What has got her husband?" we asked, as we descended the flight of steps. "He has deserted her," was the reply; and we entered the cellar. About the centre of the floor, laid upon a heap of filthy straw, was a frightfully emaciated woman, who was in the very agony of death. The only clothing she had on was a ragged chemise; and over the lower part of the body was thrown a coarse cotton sheet, beneath which also were huddled two little children, clad only in shirts, and both helpless from fever. In a corner of the cellar, a girl of about twelve years of age sat upon a scanty layer of straw. She had a tattered frock on, but no other covering, and her bare feet rested upon the damp and mouldy floor. Moving her body restlessly backwards and forwards, and with her eyes fixed on vacancy, she, also fever-stricken, was muttering incoherently to herself, and every now and then she cried out in all the wild vehemence of delirium. A broken iron pot was the only article of furniture in the place. There was not a particle of food to be seen, and, alas! there were neither cupboards nor closets to hide either "bit or sup." It would have been hard to have said whether the children or mother were most wasted from fever or starvation, but the thoughts involuntarily dwelt upon the horrible possibility of the latter. As if in bitter mockery, a bottle of medicine, duly and neatly labelled, but of which the contents had been untouched, stood upon the window-sill. The window itself was beneath the level of the street, and rags and straw usurped the place of glass, but one or two unbroken panes remaining.

As we knelt down by the side of the dying woman, the two children thrust their heads from beneath the sheet, and, gazing at us with wild and startled looks, asked for food! A deathly film had settled upon the staring eyes of the mother; and before we spoke to her, we thought that she had already escaped far beyond the reach of any sensual impression. But when, bending over her, we uttered we know not what, in a few seconds a look of intelligence struggled back to the eyes. Presently they awoke up to a full consciousness, and an amazed look stole over the face. A moment more, the eyes met ours. We spoke again, and put gently back the tangled hair from the dying woman's clammy forehead. Then it was as if the whole soul rushed back for a moment to the wan face. A radiant smile, overflowing with joy and hope, lightened the eyes and spread over the countenance; the lips slightly parted, as if they would have formed some words, but before a sound had passed between them, the light faded from the eyes, and the children were motherless. Even after death that glorious smile lingered upon the face—a smile which had winged the soul into the other world, and which had been begotten by a simple, kindly expression. Was ever doctor more richly rewarded for a mere act of duty?

If the gratitude of poor patients could be placed to a doctor's credit at his banker's, medical millionaires would be by no means rare personages. Unfortunately, however, for the profession, as much cannot be said for the more substantial thanks of our richer patients. Money cannot be so readily dispensed as thanks, and when the latter have to be transformed into a pecuniary equivalent, they often suffer sorely in the change. It is very significant of the comparative rarity of generous guerdons to the physician, that the instances on record are few in number, are worn threadbare by frequent quotation, and that few examples can be added to the stock list. Indeed, the great incomes of a few physicians are made, not by the frequent receipt of honoraria of unusual magnitude, but by incessant labour, combined with the fact that the great reputation of these men enables them at all times to claim the highest fees permitted by custom. We read of Dr. Dimsdale receiving, in 1768, from the Emperor of Russia, for inoculating the Empress and her son, a fee of 12,000*l.*, a pension for life of 500*l.* per annum, and a title of nobility; of the Emperor Joseph, of Austria, on his death-bed, making his physician Quarin a baron of the empire, and giving him an annual pension of 2000*l.*; of Sir Astley Cooper, in one instance, receiving a fee of 1000*l.*; of a provincial practitioner, since Sir Astley Cooper's time, receiving a fee of 2000*l.*; and better still, of another provincial practitioner, who very lately has been enriched by the gratitude of a patient who possessed no relatives, with a comfortable estate worth about 700*l.* per annum. We learn these things with much the same feelings as we listen to the story of the rich guerdons bestowed on the Sage Douban, in the *Arabian Nights*. We read, however, of the large incomes made at sundry times by several medical men, with very different feelings. It is pleasant to learn that Sir Astley Cooper received annually for some time 15,000*l.*, and that one year his receipts amounted to no less than 23,000*l.*; that Dr. Chambers's income reached nearly 9000*l.* per annum; and that in one particular month he pocketed 1100*l.* in fees; that Dr. Matthew Baillie one year received 11,000*l.*; and that, to speak of some of the older physicians, Dr. Mead's income from practice amounted for several years to between 5000*l.* and 6000*l.*; and Dr. Lettsom's receipts in one year reached 12,000*l.*

Dr. Fothergill's income, we may also add, averaged 6700*l.* during the last twenty-five years of his life. He, it is said, left a fortune of 80,000*l.*, yet he strove to banish "all thoughts," to use his own words, "of practising physic as a money-getting trade, with the same solicitude as he would the suggestions of vice or intemperance."

Pleasant as it may be to find that the practice of medicine does become in a few instances a very profitable pecuniary pursuit, it is by no means to be supposed that large incomes, such as we have quoted, represent, in any undue degree, generosity on the part of the public towards professors of the healing art. These incomes are the result of harassing and incessant labour of a character which the unprofessional individual cannot rightly apprehend or appreciate, and the pecuniary emolument simply represents the commercial value of the practitioner in the eyes of the public. In whatever manner we ourselves may stand upon the dignity of our profession and of honoraria, there is no doubt that the public, from whom we have to earn our livelihood, as a rule, look upon fees in the most tradesman-like fashion. In paying us, they take very great care that they will not give us more than our market value, and the income of a medical man is the index of that value with respect to the particular locality in which he resides. Although, therefore, at times a generous heart breaks through the conventionality of the established rules of payment, and is governed solely by the feelings in recompensing a medical attendant, this happens too rarely to have any permanent effect upon income derived from practice. And it is well that it should be so; for if the physician were to be dependent for his regular remuneration upon the feelings of the patient in every case, we fear he would fare badly. It must be evident that, as we have already indicated, the wide deviation of opinion which exists in the medical profession, on the proper character of the honorarium, could only lead to the utmost irregularity in practice. Add to this source of confusion those which must be found in the ordinary conception of the value and uses of money in a large mercantile community, among whom the prevalent aim will be to get whatever is required at the leastcost, and it cannot be doubted that an endless clashing of opinion would be the consequence. But custom (in this case the resultant of the combined action of a commercial atmosphere and professional worldly aspirations) stepping in, has happily prevented so sad an ending by fixing a *Zero*, in the form of a guinea, to the honorarium scale. Thus the medical man, on the one hand, is enabled to escape being victimized by recalcitrant patients; and, on the other, the public have a steady criterion to guide them in estimating the remuneration which should be tendered to him.

The public, however, rarely does otherwise than treat the question in the most business-like fashion, tendering usually the *minimum* that can be demanded from it; and at times it has recourse to trade-tricks of the dirtiest description. A capital and common "dodge" is to wheedle the physician out of his advice in the street, or club, or at the dinner-table. It answers well, and is highly economical, to get him to spend a day at your country house, and incidentally consult him about all the ailing members of your family. The substitution of a present of plate or jewelry for fees is gone out of fashion, but when it might be had recourse to, the gift of a trumpery ring (in one instance worth about four sovereigns) served capitally for an attendance extending over a period of two or three months. A fashionable French physician once remarked that, at one period of his career, he had received several presents of plate from patients, when he was unable, from want of money, to pay the expenses of his carriage. It is a coarse but very effectual trick for a single consultation, to present shillings neatly wrapped up in paper in place of a guinea. We prefer, however, the bolder expedient of coolly consulting the physician and walking off without giving a fee at all. It is not a bad idea to obtain medical aid under a false character, trusting to the known kindness of medical men. Many a substantial personage has, in this manner, defrauded the physician of his fees. Mr. Jeafferson tells of a magistrate and deputy-lieutenant for his county, ingeniously obtaining in this way a three weeks' attendance from an eminent surgeon, without paying a fee. Very recently, a lieutenant in one of our infantry regiments became a subscriber to a London hospital, and subsequently called at the house of one of the physicians to the hospital under the impression that he had a right, derived from his subscription, to consult him without fee whenever he thought fit! Not long ago, also, a country gentleman of property, unwilling to pay his ordinary medical attendant a reasonable fee for the performance of a serious surgical operation, smuggled himself into a metropolitan hospital.

It is well always to bear in mind that, if a medical man accustoms himself to demand exorbitant fees, the public have the remedy in their own hands, and they are not backward in employing it. But the medical man is almost helpless against the impositions practised upon him. These impositions, however, are perhaps trifling annoyances compared with that want of just appreciation of the nature and value of the physician's services, which is far from being uncommon, and which, perhaps, gives rise to the most provoking annoyances connected with fees. There are few things more painful to a physician than that, after attendance upon a case, the ordinary honoraria should be begrudged; yet this is not rarely the case. From the trade of the apothecary having been so long combined with the duties of a physician, in the practice of the medical men who constitute the greatest bulk of the profession, and have medical charge of by far the major portion of the population ; and from the fact that the general practitioner's remuneration has been usually sought, for many years, in the form of simple *trade* charges for medicines and mileage (the charge for medicine being made arbitrary, so that it might include a certain percentage for the intellectual element—advice), it has come to pass that the majority of individuals are accustomed to estimate the value of medical attendance by the quantity of medicine prescribed or taken. The intellectual and essential element of the physician's attendance has, indeed, been so little obtruded, that certain people have learned to look upon the "advice" as a secondary matter, and thus to regard as a grievance the being called upon to pay for it as the most important element of the treatment. This feeling, although chiefly found within the special sphere of the general practitioner, has not failed to extend its influence into all branches of medical practice. It is not a little interesting, therefore, and augurs well for the onward course of the profession as a whole, to note the change which is gradually coming over the whole system of charging among general practitioners. Moderate *fees*, so calculated as to include the cost of medicine when that is supplied, are being widely substituted for the older trade method of charging. We believe that this system has been found to work well even in practice among the poorer classes of people, due regard being given to their humble means. In proportion as the principles upon which remuneration is claimed approximate in the different branches of the profession, we may rest assured that the annoyances, arising from a wrong idea of the physician's duties, will decline, and that the whole status of the profession will rise in the eyes of the public. Whatever will tend to elevate the position of the general practitioner must powerfully contribute to this desirable end ; hence the great importance of the decision recently come to by the Royal College of Physicians, to admit general practitioners to certain rights and immunities of their College.

If, however, the medical profession would do justice to themselves in this question, it is above all things necessary that they should be true to themselves. There should be no want in each branch of the profession of a generous appreciation of the duties of the other branches. If the physician or surgeon (*pur sang*) conceives that he is at liberty, on sundry abstract notions of dignity, to treat the general practitioner with scant courtesy at the bed-side, it may be expected that from time to time such conduct will tell forcibly upon consulting fees, lowering them in a manner not very gratifying to the receiver. It is well to reflect that the general practitioner is essentially the family medical attendant of this country. It is from him that families chiefly receive their notions of the nature of medical duties; it is to him they look for guidance in those circumstances in which it may be requisite to have recourse to a physician or surgeon. He is the chief adviser as to the importance of the services which may be rendered by a physician or surgeon, the value of the time which he may have given to the care of the patient, and the reputation he has acquired. To the general practitioner, also, the physician or surgeon, on his part, has mainly to look for such information as to the worldly status of the patient, which will enable him to form his own estimate of the fee which he ought to receive for the services he has given. Apart, therefore, from the consideration of those amenities which ought to be all powerful in the association between one gentleman and another, it is a suicidal policy on the part of either physicians or surgeons to treat the general practitioner otherwise than with ordinary courtesy. Again, the omission of such courtesy has the unfortunate effect of lowering the position of the general practitioner in the eyes of his patients, and this lowering tells unfortunately not only upon the pecuniary value of his services, but is a most effective means of impeding that steady rise in the status of the whole profession which is a necessary consequence of its gradually increasing intellectual elevation. It is most unfortunate, also, and greatly to be deprecated, when the general practitioner suffers himself to be governed in his association with physicians and surgeons by petty or envious feelings, rather than by motives based upon the honour of his profession. It grates upon the feelings of the physician or surgeon, and it is simply an injustice towards him, particularly if of any standing, to be summoned with the consent of the family medical man suddenly to a consultation, perhaps at some distance from his home, or at a time when moments are to him of the greatest value, and to find that the practitioner in attendance has never broached to the family the question of the fee, or taken care that it should be of such an amount as would justly remunerate the call made upon the physician's or surgeon's time. It is not right that he should, under these circumstances, be fobbed off with the least fee that can be offered to him, unless it become a question of charity towards the patient. Most frequently, and to avoid unseemly discussion, the physician or surgeon will pocket the minimum fee and say not a word, but occasionally less nice, or indignant, about the matter, he will demand what he considers to be justly his due, and an unpleasant scene may result. If the question were one of a mere trade character, in which each person did the best he could for himself, physician and patient each striving to make the most profitable bargain, well and good; but such is not the case; and if the public generally are ever to be as thoroughly indoctrinated with a just conception of the nature of *honoraria* as the medical profession would wish, it is certain that unvarying honourable and generous conduct, one towards another, among ourselves, is requisite to the conveyance of that knowledge. Indeed, crude and false notions of the character of an *honorarium* with the public are chiefly due to shortcomings among the profession itself.

---

